# Bayesian genome-wide TWAS with reference transcriptomic data of brain and blood tissues identified 141 risk genes for Alzheimer’s disease dementia

**DOI:** 10.1186/s13195-024-01488-7

**Published:** 2024-06-01

**Authors:** Shuyi Guo, Jingjing Yang

**Affiliations:** 1 Center for Computational and Quantitative Genetics, Department of Human Genetics, Emory University School of Medicine, Atlanta, GA 30322 USA; 2 Department of Biostatistics and Data Science, School of Public Health, The University of Texas Health Science Center at Houston, Houston, TX 77030 USA

**Keywords:** Bayesian genome-wide TWAS, Alzheimer’s disease dementia, *Cis*-eQTL, *Trans*-eQTL, Aggregated cauchy association test

## Abstract

**Background:**

Transcriptome-wide association study (TWAS) is an influential tool for identifying genes associated with complex diseases whose genetic effects are likely mediated through transcriptome. TWAS utilizes reference genetic and transcriptomic data to estimate effect sizes of genetic variants on gene expression (i.e., effect sizes of a broad sense of expression quantitative trait loci, eQTL). These estimated effect sizes are employed as variant weights in gene-based association tests, facilitating the mapping of risk genes with genome-wide association study (GWAS) data. However, most existing TWAS of Alzheimer's disease (AD) dementia are limited to studying only *cis*-eQTL proximal to the test gene. To overcome this limitation, we applied the Bayesian Genome-wide TWAS (BGW-TWAS) method to leveraging both *cis*- and *trans*- eQTL of brain and blood tissues, in order to enhance mapping risk genes for AD dementia.

**Methods:**

We first applied BGW-TWAS to the Genotype-Tissue Expression (GTEx) V8 dataset to estimate *cis*- and *trans*- eQTL effect sizes of the prefrontal cortex, cortex, and whole blood tissues. Estimated eQTL effect sizes were integrated with the summary data of the most recent GWAS of AD dementia to obtain BGW-TWAS (i.e., gene-based association test) *p*-values of AD dementia per gene per tissue type. Then we used the aggregated Cauchy association test to combine TWAS *p*-values across three tissues to obtain omnibus TWAS *p*-values per gene.

**Results:**

We identified 85 significant genes in prefrontal cortex, 82 in cortex, and 76 in whole blood that were significantly associated with AD dementia. By combining BGW-TWAS *p*-values across these three tissues, we obtained 141 significant risk genes including 34 genes primarily due to *trans*-eQTL and 35 mapped risk genes in GWAS Catalog. With these 141 significant risk genes, we detected functional clusters comprised of both known mapped GWAS risk genes of AD in GWAS Catalog and our identified TWAS risk genes by protein-protein interaction network analysis, as well as several enriched phenotypes related to AD.

**Conclusion:**

We applied BGW-TWAS and aggregated Cauchy test methods to integrate both *cis*- and *trans*- eQTL data of brain and blood tissues with GWAS summary data, identifying 141 TWAS risk genes of AD dementia. These identified risk genes provide novel insights into the underlying biological mechanisms of AD dementia and potential gene targets for therapeutics development.

**Supplementary Information:**

The online version contains supplementary material available at 10.1186/s13195-024-01488-7.

## Background

Alzheimer's disease (AD) dementia is a complex neurodegenerative disorder characterized by progressive cognitive decline and memory loss, currently affecting ~7 million Americans aged 65 and older. AD dementia is listed as the seventh-leading cause of death in the United States of America in 2021 [1]. Despite extensive research, the underlying biological mechanisms of AD dementia remain elusive, and effective treatments are still lacking [2]. Recent studies have highlighted the important roles of genomic risk factors of AD dementia [3, 4]. Recent genome-wide association studies (GWAS) identified a total of ~75 genetic risk loci for AD dementia [5]. However, these risk loci still only explain a small portion of the heritability of AD dementia, suggesting additional risk genes or loci might contribute to the genetic etiology of AD dementia. Also, the biological mechanisms underlying the majority of the mapped risk genes remain unknown. Transcriptome-wide association study (TWAS) has emerged as an influential tool for identifying risk genes associated with complex diseases, particularly those with genetic effects mediated through transcriptome [6, 7]. For example, the recent TWAS of AD dementia by Sun et al. identified 53 risk genes by standard two-stage TWAS methods [8].

Standard two-stage TWAS methods first train gene expression prediction models by using reference genetic and transcriptomic data profiled of the same training samples, taking quantitative gene expression traits as response variables and the proximal *cis*-acting genetic variants as predictors (Stage I). Estimated effect sizes of genetic variants in the gene expression prediction models could be viewed as effect sizes of a broad sense of expression quantitative trait loci (eQTL), which will then be taken as variant weights in gene-based association test to map risk genes with GWAS data (Stage II). TWAS association test in Stage II is equivalent to testing the association between predicted genetically regulated gene expression (GReX) levels and the phenotype of interest in the GWAS data [9].

However, one limitation of most existing TWAS methods is that they only consider *cis*-eQTL [6, 10, 11], i.e., proximal genetic variants (e.g., located within the 1Mb region around the target gene region), in the gene expression prediction models. The limitation is mainly due to the computational bottleneck of considering genome-wide genetic variants to fit gene expression prediction models for transcriptome-wide ~20K genes per tissue type. The resulting cavity is failing to account for *trans*-eQTL, i.e., located distal from the target gene region, that have been found playing important roles in biological processes and disease susceptibility [12, 13]. Incorporating *trans*-eQTL in TWAS is essential as they can reveal additional regulatory mechanisms, help identify additional risk genes, and further our understanding of the underlying biological mechanisms of complex diseases.

To overcome this limitation in studying AD dementia, we employed the Bayesian Genome-wide TWAS (BGW-TWAS) method that incorporated both *cis*- and *trans*-eQTL for TWAS [14]. We applied BGW-TWAS to the reference Genotype-Tissue Expression (GTEx) V8 dataset [15] of three tissues –– prefrontal cortex, cortex, and whole blood. The selection of prefrontal cortex and cortex tissues was based on substantial evidence linking their involvement to the progression of AD dementia [16]. The selection of whole blood tissue was due to three reasons: i) a large sample size exists (*n*=574) for the whole blood tissue in the reference GTEx V8 dataset; ii) gene expression in the whole blood and that of the brain's cortex were found correlated [17]; iii) recent studies have demonstrated that gene expressions in whole blood could serve as biomarkers for AD dementia [18, 19]. For example, multiple studies showed that blood-based transcriptomics biomarkers predict AD risks, such as in Korukonda et. al. Alzheimer’s & Dementia 2021 [20]; Shigemizu et. al. Alzheimer’s Research & Therapy 2020 [21], Abdullah et. al. Informatics in Medicine 2022 [22],and Lee et. al Scientific Reports 2020 [23]. Also, a recent paper by Angioni et. al. J Prev Alzheimers Dis. 2022 [24] discussed the success, challenges, and future directions of deriving blood biomarkers from research use to clinical practice.

**Fig. 1:**
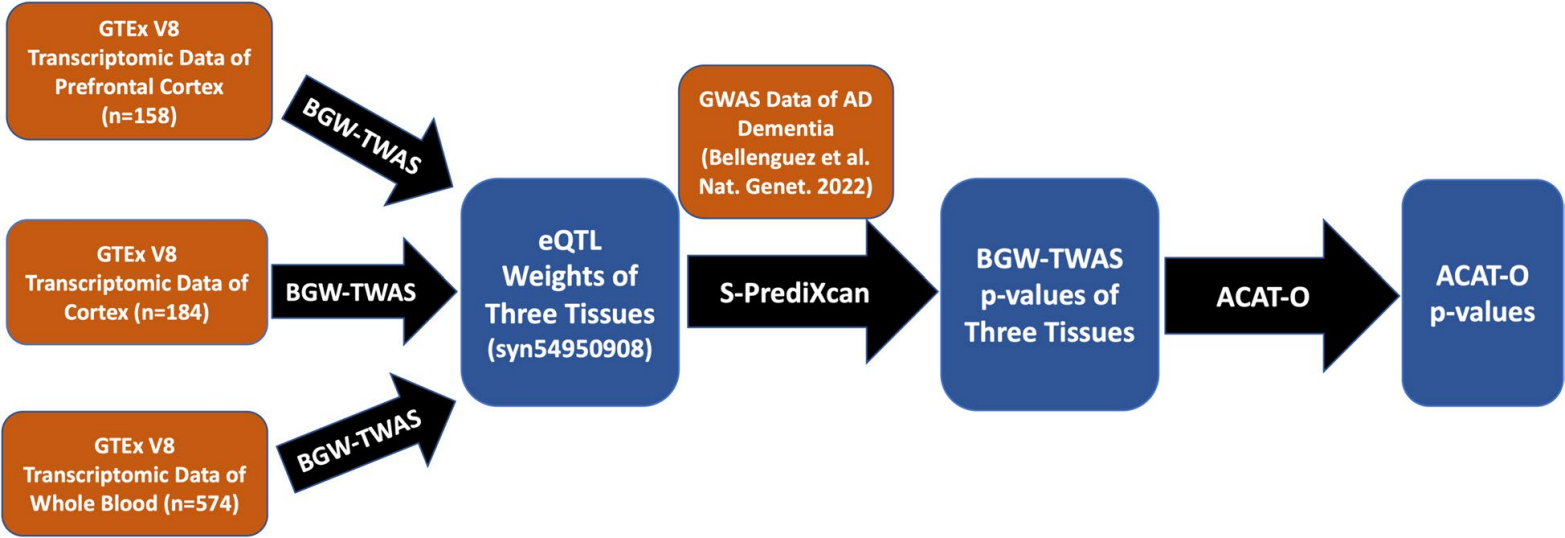
Study workflow

In this study, we first estimated *cis*- and *trans*- eQTL effect sizes of prefrontal cortex, cortex, and whole blood tissues by BGW-TWAS. Second, we calculated BGW-TWAS *p*-values using the S-PrediXcan test statistic (gene-based association test) [25], where the estimated eQTL effect sizes were integrated with the summary data of the most recent GWAS of AD dementia (*n*=~487K) [26]. BGW-TWAS *p*-values were obtained for transcriptome-wide test genes for each tissue. Third, for each test gene, we used the omnibus aggregated Cauchy association test (ACAT-O) [27, 28] to combine TWAS *p*-values across three tissues to obtain the omnibus TWAS *p*-value. The workflow is presented in Fig. 1.

As a result, we identified 85 significant genes with the prefrontal cortex, 82 significant genes with the cortex, and 76 significant genes with the whole blood reference transcriptomic data. Combining BGW-TWAS *p*-values across these three tissues by ACAT-O, we obtained a total of 141 significant TWAS risk genes for AD dementia, including several well-known AD risk genes and risk genes that not mapped in the GWAS Catalog. Through protein-protein interaction network analysis [29] with these 141 significant TWAS risk genes, we detected gene networks comprised of known AD risk genes such as *APOC2*, *BIN1*, *CASS4*, *MS4A4A*, *MS4A6A*, *SLC24A4,* and *MAPT* and additional risk genes that are not mapped in the GWAS Catalog*.* In addition, these 141 significant TWAS risk genes were found enriched with known risk genes of several AD related phenotypes.

## Methods

### Bayesian genome-wide TWAS (BGW-TWAS)

BGW-TWAS [14] is a recently proposed TWAS method that incorporates both *cis*- and *trans*- genetic variants of the target genes as predictors in the gene expression prediction models (Stage I) as follows:

1 $${\mathcal{E}}_{g}={{\varvec{X}}}_{cis}{{\varvec{w}}}_{cis}+{{\varvec{X}}}_{trans}{{\varvec{w}}}_{trans}+{\varvec{\epsilon}}, {\epsilon }_{i}\sim N\left(0, {\sigma }_{\epsilon }^{2}\right),$$

where ${\mathcal{E}}_{g}$ denotes the target gene expression quantitative trait, (${{\varvec{X}}}_{cis}, {{\varvec{X}}}_{trans}$) denotes the genotype data matrix of *cis*- and *trans*- genetic variants; (${{\varvec{w}}}_{cis}$, ${{\varvec{w}}}_{trans}$) denotes the corresponding *cis*- and *trans*- effect sizes; and ${\varvec{\epsilon}}$ denotes the errors following a normal distribution with mean 0 and variance ${\sigma }_{\epsilon }^{2}$. Here, we briefly describe the BGW-TWAS method.

BGW-TWAS assumes the Bayesian variable selection regression (BVSR) model [30] to enforce sparse eQTL models, by assuming specific spike-and-slab prior distributions for *cis*- and *trans*- effect sizes given by

2 $$\begin{array}{c}{w}_{cis,i} \sim {\pi }_{cis}N\left(0, {\sigma }_{cis}^{2}{\sigma }_{\epsilon }^{2}\right)+\left(1-{\pi }_{cis}\right){\delta }_{0}\left({w}_{cis,i}\right); \\ {w}_{trans,i} \sim {\pi }_{trans}N\left(0, {\sigma }_{trans}^{2}{\sigma }_{\epsilon }^{2}\right)+\left(1-{\pi }_{trans}\right){\delta }_{0}\left({w}_{cis,i}\right).\end{array}$$

Here, $({\pi }_{cis}, {\pi }_{trans})$ denote the respective probability that the corresponding effect size is normally distributed, and ${\delta }_{0}\left({w}_{i}\right)$ is the point mass density function that takes value 0 when ${w}_{i}\ne 0$ and 1 when ${w}_{i}=0$.

BGW-TWAS employs multiple computational strategies to enable efficient computation to account for genome-wide genetic variants as predictors in the gene expression prediction models as in Eq. 1. A previously developed scalable expectation-maximization Markov Chain Monte Carlo (EM-MCMC) algorithm [31] is adapted and used by BGW-TWAS. Genome-wide variants are first segmented into approximately independent genome blocks. Genome blocks containing *cis*- genetic variants or containing top *trans*-eQTL with single variant test *p*-values < ${10}^{-5}$ will be selected for implementing the EM-MCMC algorithm to estimate eQTL effect sizes in the joint multiple regression model as in Eq. 1. The posterior causal probabilities (PCP) for “eQTL” with non-zero posterior effect size estimates will also be estimated. The product of estimated PCPs and effect sizes will represent the expected posterior effect sizes and be used as variant weights in the follow-up gene-based association tests (Stage II). The details of the BGW-TWAS method are referred to the BGW-TWAS paper [14].

The BGW-TWAS tool is memory efficient for first generating single variant eQTL summary data with individual-level reference transcriptomic and genetic data, and then using only these summary eQTL data in the MCMC algorithm. Also, the BGW-TWAS tool implements MCMC algorithm in parallel for multiple genome blocks, which can best utilize high-performance computing clusters with multiple computation cores. In this study, the BGW-TWAS method finished analyzing an average gene with sample size 184 in the reference transcriptomic dataset within 5 minutes in 16 cores. A total of 16 Gb memory was requested and sufficient for all analyses in this study.

### Gene-based association test by S-PrediXcan test statistic

With *cis*- and *trans*- eQTL weights estimated by BGW-TWAS method and summary-level GWAS data (i.e., Z-scores by single variant tests), we employed the S-PrediXcan [25] approach to calculate the burden type TWAS Z-score test statistic ${Z}_{g}$ per gene as follows:

3 $$\begin{array}{c}{Z}_{g}=\sum_{l\in {\text{ Model}}_{g}} \widetilde{{\text{w}}_{\text{lg }}}\frac{\widehat{{\sigma }_{l}}}{\widehat{{\sigma }_{g}}}\frac{\widehat{{\beta }_{l}}}{SE\left(\widehat{{\beta }_{l}}\right)}=\sum_{l\in {\text{ Model}}_{g}} \widetilde{{\text{w}}_{\text{lg }}}\frac{\widehat{{\sigma }_{l}}}{\widehat{{\sigma }_{g}}}{Z}_{l}=\sum_{l\in {\text{ Model}}_{g}} \frac{\left(\widetilde{{\text{w}}_{\text{lg }}}\widehat{{\sigma }_{l}}\right){Z}_{l}}{\sqrt{{\widehat{w}}^{\,\prime}{\varvec{V}}\widehat{w}}},\\ \widehat{{\sigma }_{l}^{2}}=\text{Var}\left({x}_{l}\right), \widehat{{\sigma }_{g}^{2}}={\widetilde{{\varvec{w}}}^{\,\prime}}V\widetilde{{\varvec{w}}}, V=Cov({{\varvec{X}}}_{{\varvec{r}}{\varvec{e}}{\varvec{f}}}),\end{array}$$

where $\widehat{{\beta }_{l}}$ denotes the genetic effect size of variant $l$ from GWAS, ${Z}_{l}$ denote the corresponding Z-score statistic by single variant GWAS test, and $\widetilde{{\text{w}}_{\text{lg }}}=\widehat{P{CP}_{l}}{\widehat{w}}_{l}$ is the eQTL weight of variant $l$ estimated by BGW-TWAS. Here, ${{\varvec{X}}}_{{\varvec{r}}{\varvec{e}}{\varvec{f}}}$ denotes the genotype matrix with genotype covariance matrix ${\varvec{V}}$ from a reference panel. Two-tailed BGW-TWAS *p*-values can then be obtained from the TWAS Z-score test statistics as in Eq. 3.

### Omnibus aggregated cauchy association test (ACAT-O)

ACAT-O is an omnibus test that can combine *p*-values of multiple tests with respect to the same null hypothesis [27, 28], which employs a linear combination of transformed *p*-values as the test statistic. Particularly, the ACAT-O method is a general statistical method, which is suitable for combining correlated *p*-values such as the ones generated with correlated transcriptomic data of multiple tissues. As shown in the following formula,

4 $${T}_{ACAT-O}=\frac{1}{K}\sum_{i=1}^{K} \text{tan}\left\{\left(0.5-{p}_{i}\right)\pi \right\},$$

where $\{{p}_{i}, i=1,\dots , K\}$ denotes the *p*-values of K tests and the ACAT-O statistic ${T}_{ACAT-O}$ approximately follows a standard Cauchy distribution under the null hypothesis. The approximate standard Cauchy distribution allows analytical calculations of the ACAT-O *p*-values, which were shown to be more accurate for combining small/significant *p*-values [27, 28].

As shown in Fig. 1, in this study, ACAT-O method was used to combine BGW-TWAS *p*-values (per gene) across three considered tissues to obtain combined TWAS *p*-values, under the null hypothesis of the combined genetic effects are mediated through the transcriptome of one of these three tissues. Combined TWAS *p*-values were obtained by using the “ACATO” function from the R package “sumFREGAT” [32].

Since the ACAT-O method only provides an omnibus combined *p*-value for each test gene, one could refer to the TWAS Z-score test summary data of each tissue to gain information about which tissue contributed most to the significant TWAS risk gene by ACAT-O.

### Genotype-tissue expression (GTEx) data

The publicly available GTEx V8 (dbGaP phs000424.v8.p2) data [15] contain whole genome sequencing (WGS) data of 838 human donors and RNA sequencing (RNA-seq) transcriptomic data of 17,382 normal samples from 52 human tissues and two cell lines. We use the transcriptomic data of the prefrontal cortex (*n*=158), cortex (*n*=184), and whole blood (*n*=574) tissues and the corresponding WGS data as reference panel to estimate *cis*- and *trans*- eQTL weights by BGW-TWAS. Samples in the GTEx V8 dataset are of European ancestries. The same fully processed, filtered, and normalized transcriptomic data used in the GTEx eQTL analysis were downloaded from the GTEx portal and use in this study. For each tissue, samples with <10 million mapped RNA-seq reads were excluded. For samples with replicates, the replicate with the greatest number of reads was selected. Gene read counts from each sample were normalized using size factors calculated by DESeq2 and log-transformed with an offset of 1. Genes with log-transformed value $>1$ in $>10\%$ samples were considered. The resulting gene expression values were centered with mean 0 and standardized with standard deviation 1. The resulting matrix was then hierarchically clustered (based on average and cosine distance), and a chi2 *p*-value was calculated based on Mahalanobis distance. Clusters with ≥60% samples with Bonferroni-corrected *p*-values <0.05 were marked as outliers, and their samples were excluded. Genetic variants with missing rate $<20\%$, minor allele frequency $>0.01$, and Hardy-Weinberg equilibrium *p*-value $>{10}^{-5}$ were considered for fitting the gene expression prediction models.

The fully processed, filtered, and normalized transcriptomic data were adjusted for top five genotype principal components, top probabilistic estimation of expression residuals (PEER) factors [9], sequencing protocol (PCR-based or PCR-free), sequencing platform (Illumina HiSeq 2000 or HiSeq X), and sex, as suggested by the GTEx eQTL data analysis guidelines [15]. The number of top PEER factors [33] used to adjust the gene expression traits depends on the sample sizes in the reference transcriptomic data cohort. We used 30 factors for gene expression traits of the prefrontal cortex and cortex tissues, and 60 factors for the whole blood tissue. Only samples with complete data of these covariates were included in the analyses. Adjusted gene expression quantitative traits were then taken as response variables in the gene expression prediction model.

### Summary-level GWAS data of AD dementia

The summary-level GWAS data of AD dementia (i.e., single variant Z-score test statistics obtained by meta-analysis) were generated by the latest GWAS by Bellenguez et al. [5]. The summary-level GWAS data (*n*=~487K; European ancestry) were generated by meta-analysis with the European Alzheimer & Dementia Biobank consortium (with samples from 15 European countries) and UK Biobank dataset. GWAS of each cohort was conducted with clinically diagnosed cases, ancestry proxy cases, and controls.

### Protein-protein interaction network and pathway analysis

STRING (version 12.0) [34] is a bioinformatics web tool that provides information on protein-protein interactions and networks, as well as functional characterization of genes and proteins. The tool integrates different types of evidence from public databases, such as genomic context, high-throughput experiments, and previous knowledge from other databases, to generate reliable predictions of protein interactions and build networks and pathways. Provided with a list of gene names, STRING will construct networks based on the protein-protein interactions of the corresponding proteins, as well as identify phenotypes that have risk genes enriched in the provided list. Proteins corresponding to provided genes are considered as nodes in the protein-protein interaction network. Protein-protein edges represent the predicted functional associations, colored differently to indicate seven categories –– computational interaction predictions from co-expression, “text-mining” of scientific literature, databases of interaction experiments (biochemical/genetic data), known protein complexes or pathways from curated resources, gene co-occurrence, gene fusion and gene neighborhood. Gene co-occurrence, fusion, and neighborhood represent association predictions are based on whole-genome comparisons [35]. Interactions from these resources are critically assessed, scored, and subsequently automatically transferred to less well-studied organisms using hierarchical orthology information [34].

Particularly, the “text-mining” channel is the result of parsing full-text articles from the PMC Open Access Subset (up to April 2022), PubMed abstracts (up to August 2022), as well as summary texts from OMIM [36] and Saccharomyces genome database [37] entry descriptions. These texts are all parsed for co-mentions of protein pairs and assessed against the frequencies of all separate mentions of the respective proteins. An improved deep learning-based relation extraction text mining model was used by STRING v12 [34]. The ‘textmining’ channel significantly increases the number of protein–protein interactions.

## Results

### BGW-TWAS results of AD dementia

Using BGW-TWAS method, we trained gene expression prediction models for 23,724 genes of prefrontal cortex tissue, 23,900 genes of cortex tissue, and 19,519 genes of whole blood tissue. We identified 85, 82, and 76 TWAS risk genes with significant *p*-values (with Bonferroni correction) for prefrontal cortex, cortex, and whole blood tissues, respectively. Of these, 20 genes were significant for prefrontal cortex and cortex, 4 were significant for prefrontal cortex and whole blood, 5 were significant for cortex and whole blood, and 13 were significant for all three tissue types (Supplemental Figure 1). Detailed information of the significant TWAS risk genes of these three tissues were provided in Supplemental Files 2-4. Manhattan plots of BGW-TWAS results of these three tissues were presented in Supplemental Figures 2-4.

We also summarized the proportion of *trans*-eQTL with non-zero weights for all significant genes of three tissues in Supplemental Figure 5. There were 27 (31.8%), 19 (23.2%), and 21 (27.6%) significant genes whose association were driven by ≥50% *trans*-eQTLs in prefrontal cortex, cortex, and whole blood tissues, respectively. These results demonstrated that *trans*-eQTL had important contributions to significant TWAS risk genes of all three tissues.

### ACAT-O results of AD dementia

**Fig. 2:**
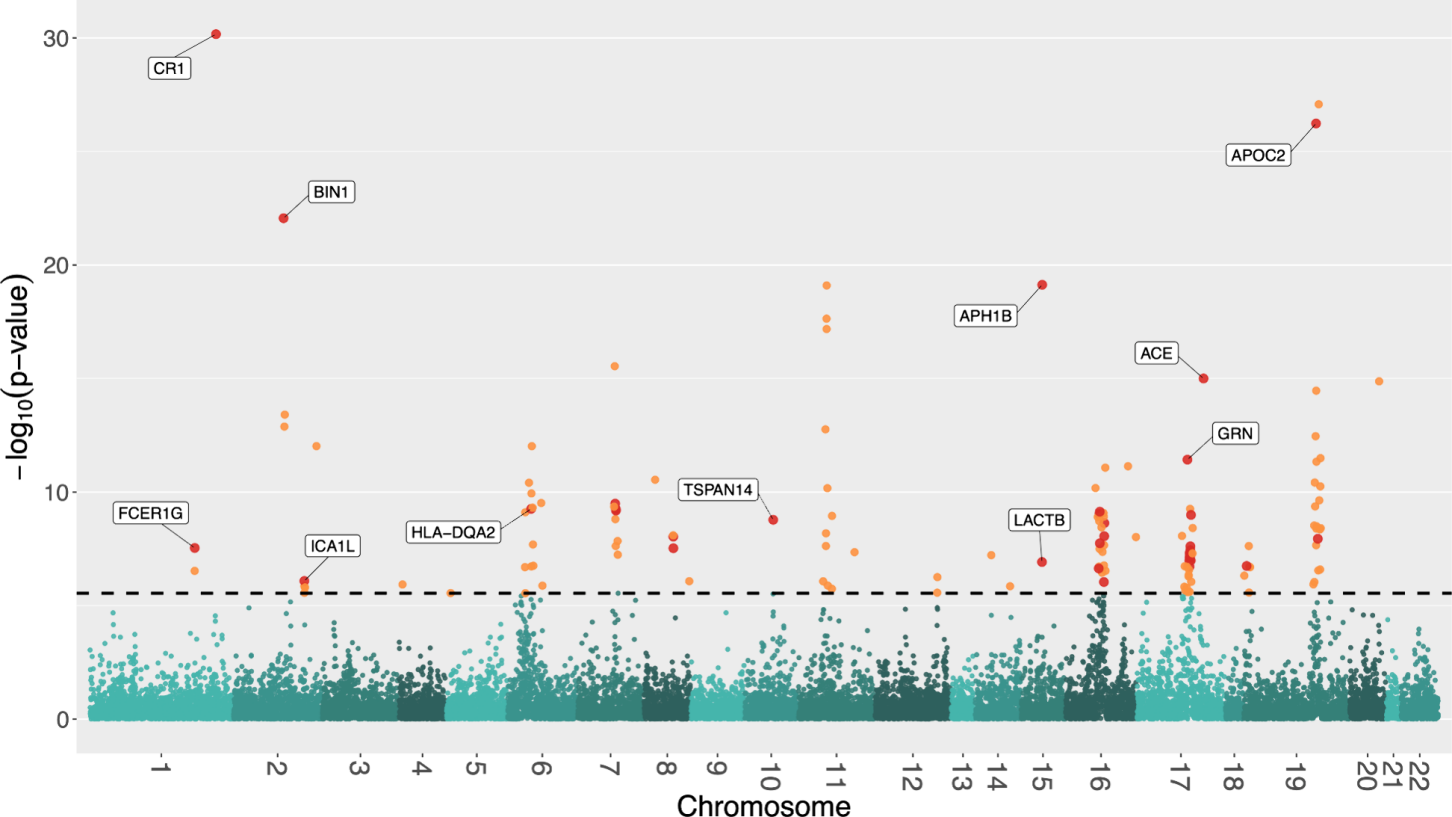
Manhattan plot of ACAT-O TWAS *p*-values for studying AD dementia. The horizontal dashed line represents the Bonferroni corrected significance threshold of the *p*-values. Orange dots indicate genes that were significant in only one tissue, while red dots highlight genes that were significant in more than one tissue

Combining BGW-TWAS *p*-values across three tissues by ACAT-O, we obtained ACAT-O *p*-values for a total of 17,449 genes. We identified 141 genes with significant ACAT-O *p*-values (with Bonferroni correction). As illustrated by the Manhattan plot (Fig. 2), 27 genes were located on chromosome 19 around the well-known *APOE* locus, which is consistent with prior TWAS findings [8, 38].

To compare with TWAS results using only *cis*-eQTL, we conducted TWAS for all three tissue types using only *cis*-eQTL with non-zero weights estimated by BGW-TWAS, and then combined these TWAS *p*-values per gene by ACAT-O. We found that 107 of the 141 significant genes (using both *cis*- and *trans*- eQTL) retained their significance using only *cis*-eQTL, while the remaining 34 genes were not detected (Supplemental Figure 6). Because these 34 (~24%) significant genes were primarily driven by *trans*-eQTL. We presented example significant TWAS risk genes (63 out of all 141) in Table 1, including these 34 significant genes driven by *trans*-eQTL and additional 29 risk genes that were significant in both prefrontal cortex and cortex tissues. Summary data of all significant TWAS risk genes were presented in the Supplemental File 1.

**Table 1:** Example TWAS risk genes of AD dementia with significant ACAT-O *p*-values, which were mainly driven by *cis*-eQTL and significant in both prefrontal cortex and cortex tissues (29 in the left column) or *trans*-eQTL (35 in the right column). TWAS risk genes driven by *trans-*eQTL were only found significant in one tissue, demonstrating tissue-specificity

**TWAS risk genes driven by *cis*-eQTL**			**TWAS risk genes driven by *trans*-eQTL**		
**Gene**	**CHR**	**ACAT-O** ***p*-value**	**Gene**	**CHR**	**ACAT-O** ***p*-value**
CR1^ab^	1	6.85E-31	NRP2^a^	2	1.60E-06
ICA1L^ab^	2	8.25E-07	HAUS3^a^	4	1.18E-06
HLA-DQA2^ab^	6	5.49E-10	GTF2H4^b^	6	2.86E-06
PVRIG^ab^	7	6.56E-10	CUL7^b^	6	1.33E-06
STAG3^ab^	7	5.66E-10	TUBB^b^	6	2.03E-07
AP4M1^ab^	7	3.23E-10	DDR1^a^	6	7.83E-10
NDUFAF6^ab^	8	2.98E-08	TRERF1^c^	6	3.04E-10
TSPAN14^ab^	10	1.67E-09	SNORD22^a^	11	1.83E-06
LACTB^abc^	15	1.21E-07	VWCE^b^	11	1.32E-06
SEZ6L2^ab^	16	2.31E-07	SLC3A2^c^	11	1.13E-09
PRSS36^ab^	16	2.33E-09	AP001350.4^b^	11	1.74E-13
INO80E^abc^	16	9.48E-10	C16orf58^a^	16	2.95E-07
YPEL3^abc^	16	7.34E-10	SLC7A5P1^c^	16	1.26E-09
ARHGAP27^ab^	17	1.93E-07	RP11-196G11.3^a^	16	1.21E-09
ARL17A^ab^	17	1.04E-07	NUPR1^c^	16	6.68E-11
PLEKHM1^abc^	17	7.82E-08	TGFB1I1^b^	16	8.45E-12
DND1P1^abc^	17	5.87E-08	EFTUD2^b^	17	2.56E-06
LRRC37A2^abc^	17	5.83E-08	NBR1^a^	17	2.17E-06
MAPK8IP1P2^abc^	17	4.89E-08	C17orf53^a^	17	1.63E-06
LINC02210^abc^	17	4.84E-08	AOC2^c^	17	1.49E-06
LRRC37A4P^abc^	17	4.78E-08	RP5-882C2.2^a^	17	1.98E-07
MAPK8IP1P1^abc^	17	3.68E-08	RUNDC1^c^	17	1.86E-07
RP11-259G18.3^abc^	17	2.45E-08	SP2^c^	17	5.04E-08
WNT3^abc^	17	1.02E-09	RAB5C^a^	17	8.57E-09
GRN^ab^	17	3.75E-12	SP2-AS1^a^	17	3.84E-09
ACE^abc^	17	9.99E-16	CADM4^a^	19	1.16E-06
POLR2E^ab^	19	1.81E-07	PRKD2^b^	19	2.59E-07
GPR4^ab^	19	1.15E-08	DOT1L^a^	19	2.03E-07
APOC2^ab^	19	5.87E-27	CTB-12A17.2^a^	19	4.45E-09
a: Genes significant in prefrontal cortex b: Genes significant in cortex c: Genes significant in whole blood			TMEM160^c^	19	3.91E-09
			PPP5C^a^	19	2.33E-10
			SLC1A5^c^	19	5.63E-11
			ZNF226^c^	19	3.82E-11
			CTD-2233K9.1^a^	19	3.23E-12

### Contributing eQTL of significant TWAS risk genes

**Fig. 3:**
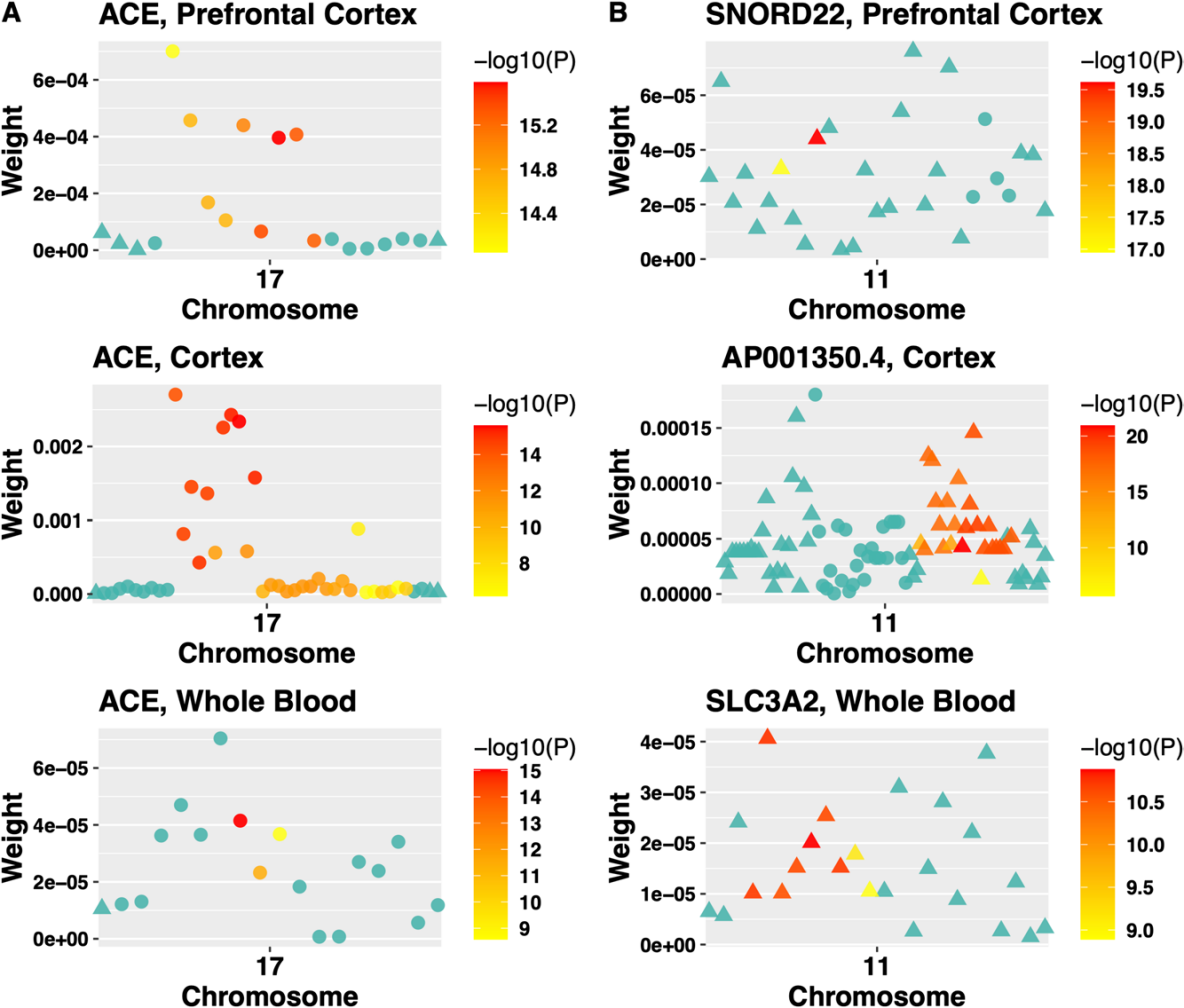
Scatter plots of eQTL weights estimated by BGW-TWAS of example TWAS risk genes. Column A: gene *ACE* (chr17) in three tissues; Column B: gene *SNORD22* (chr11), *AP001350.4* (chr11)*,* and *SLC3A2* (chr11), in prefrontal cortex, cortex, and whole blood tissues, respectively. Y-axis depicts the values of eQTL weights estimated by BGW-TWAS, and the x-axis shows the order of base pair position of the corresponding eQTL. Solid circles denote *cis*-eQTL, and triangles refer to *trans*-eQTL. Color legend denotes the -log (GWAS *p*-value) of the corresponding eQTL

To investigate how eQTL contributed to significant TWAS risk genes, we plotted eQTL weights estimated by BGW-TWAS for three tissues of example TWAS risk genes in Fig. 3. Specifically, column A in Fig. 3 shows the eQTL weights (in three tissues) of gene *ACE* whose significance is primarily due to *cis*-eQTL (circles); and column B shows the eQTL weights of three genes (*SNORD22*, *AP001350.4*, and *SLC3A2*) whose significance is primarily due to *trans*-eQTL (triangles) in prefrontal cortex, cortex, and whole blood tissues, respectively. As shown in Fig. 3, each dot represents one eQTL with colors ranging from yellow to red to represent the corresponding AD dementia GWAS *p*-value. We can see that *cis*- or *trans*- eQTL colocalizing with potential significant GWAS *p*-values (colored from yellow to red) are driving the significant TWAS association of the test gene.

It is noticeable that all the *trans*-eQTL of the example genes (and for most of the genes we studied) are still on the same chromosome as the test gene. This might be due to both biological mechanisms and methodological limitations. Because the BGM-TWAS method assumes a sparse eQTL model for gene expression and often can only estimate eQTL with relatively large effect sizes, it is possible that *trans*-eQTL located on the same chromosome as the test gene have relatively larger effect sizes. Thus, the BGW-TWAS method only estimated non-zero weights for these *trans*-eQTL that were used in the follow-up gene-based association studies.

### Known mapped GWAS risk genes in GWAS catalog for AD dementia

Compared to previous GWAS results, 35 out of the 141 TWAS risk genes were reported as mapped risk genes of AD dementia in GWAS Catalog [39], such as known risk genes of *ACE*, *APOC2*, *BIN1*, and *CR1* (Supplemental File 1). Particularly, the GReX of *CR1 and APOC2* were both found to be negatively associated with risk of AD dementia in prefrontal cortex and cortex. The GReX of *BIN1* was positively associated with risk of AD dementia in both cortex and whole blood tissue. The GReX of *ACE* was positively associated with risk of AD dementia in all three tissues. These findings are consistent with previous studies about genes *CR1 *[40]*, APOC2 *[41]*, BIN1 *[42], and *ACE* [43]. Information of upregulation and downregulation of the complete lists of significant genes in the 3 tissues is provided in the Supplemental Files 2-4.

### Additional TWAS risk genes of AD dementia

Besides these 35 mapped risk genes reported in GWAS catalog, we identified additional 106 significant TWAS risk genes of AD dementia, including 34 genes whose significance were primarily due to *trans*-eQTLs. Also, our findings replicated risk genes identified by recent studies of AD that integrated eQTL data with GWAS summary data. For instance, genes *OSBP*, *ZNF296,* and *ZNF284* were identified by the TWAS tool TIGAR [9, 11] using only *cis*-eQTL of the prefrontal cortex tissue [44] and GWAS summary data of AD dementia generated by Wightman D.P. et. al in 2021 [26]. A recent study integrating summary-level GWAS and meta-analytic *cis*-eQTL data found genes *NDUFS2* and *PRSS36* were related to AD risk [45]. Recent splicing TWAS analyses found that causal splicing introns of gene *WDR33* and *LRRC37A4P* were associated with AD in multiple brain tissues [46]. A recent study that integrated eQTL data of blood tissue and GWAS of late-onset AD (LOAD) by a Bayesian statistical method identified risk gene *ZNF226* [47].

We also looked at *cis*-SNPs within ±1Mb region of our identified TWAS risk genes, using the GWAS results by Bellenguez et al. [5]. We found that 114 genes have at least one *cis*-SNP with GWAS *p*-value less than the genome-wide significance threshold (5E-8) and 27 genes have no GWAS significant *cis*-SNPs (Supplemental File 1). Because our significant TWAS risk genes were identified by testing both *cis*- and *trans*-SNPs with non-zero eQTL weights. Those genes without GWAS significant *cis*-SNPs will not be identified by standard GWAS with the same data. Our findings could be due to the consideration of *trans*-eQTL and/or burden tests with multiple eQTL. Previous biological studies provided insights of some of our findings with no significant *cis*-SNPs. For example, it was found that the expression of *NRP2*, a gene coding the modulating receptors of the vascular endothelial growth factor signaling family, was associated with better cognitive outcomes [48]. Gene *OTULIN* was found to affect NF-κB-activity in AD patients, subsequently leading to the shrinkage of the entorhinal cortex and the limbic system in early stages of AD [49]. Gene *DDR1* is associated with reduced inflammation and vascular fibrosis in AD [50]. In addition, gene *TUBB* encodes a β-tubulin protein that forms a dimer with alpha tubulin and acts as a structural component of microtubules, where β-tubulin was found aggregating in AD cases [51].

### Protein-protein interaction network analysis by STRING

**Fig. 4:**
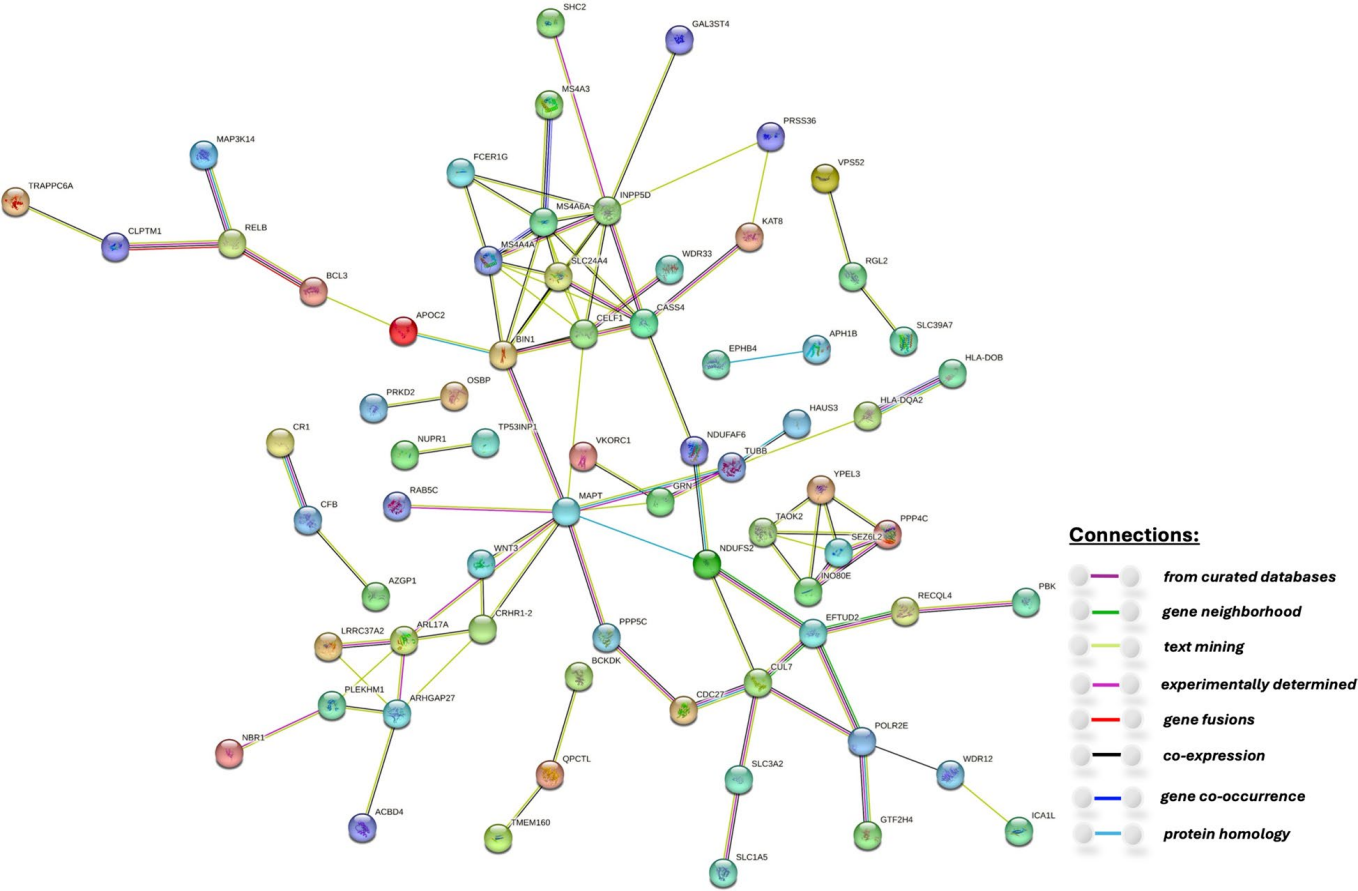
Protein-protein interaction networks identified with 141 significant TWAS risk genes by the STRING tool. Edge colors show different sources of the identified protein-protein interactions

To further understand the underlying biological mechanisms of our identified 141 TWAS risk genes, we constructed protein-protein interaction networks and performed phenotype enrichment analysis using the STRING tool. As shown in Fig. 4, we identified several clusters of genes, including a major cluster composed of known mapped GWAS risk genes of AD such as *BIN1*, *CASS4*, *MS4A4A*, *MS4A6A*, *MS4A3*, *SLC24A4*, *INPP5D*, *FCER1G*, *APOC2*, *MAPT*, *BCL3*, *RELB*, and *TRAPPC6A*. Importantly, this major gene network is connected through well-known risk genes *APOC2*, *BIN1*, and *MAPT*. Especially, *APOC2* is known to be related with both lipids and AD [52]; *BIN1* is a key regulator of proinflammatory and neurodegeneration-related activation in microglia [53]; and *MAPT* encodes the microtubule-associated protein tau (MAPT) which is known as a key AD pathology [54]. Additionally, several of our findings that are not reported in GWAS Catalog and/or have no *cis*-SNP with significant GWAS *p*-value were found connected with these known AD risk genes in the main cluster. For example, gene *WDR33* was connected to *CELF1*; genes *SHC2* and *GAL3ST4* were connected to *INPP5D*; genes *WNT3*, *PPP5C* and *NDUFS2* were connected to *MAPT*.

By pathway enrichment analyses conducted by the STRING tool, five pathways were found to be enriched with our identified TWAS risk genes. Three of these pathways were involved with the CD20-like family such as Cranial nerve maturation, Peptidase A22B, signal peptide peptidase and GTPase GIMA/IAN/Toc. These three pathways included the key known AD risk genes *MS4A4A*, *SLC24A4*, *MS4A6A*, *BIN1*, and *CASS4*. The fourth pathway was ADP-ribosylation factor-like protein 17, and LRRC37A/B like protein 1, C-terminal, which included TWAS risk genes *LRRC37A2, ARHGAP27,* and *ARL17A* on chromosome 17. The fifth pathway was Cranial nerve maturation, and His Kinase A (phosphoacceptor) domain, including known AD risk genes *SLC24A4*, *CASS4*, and *BIN1*.

### Phenotype enrichment analysis

**Fig. 5:**
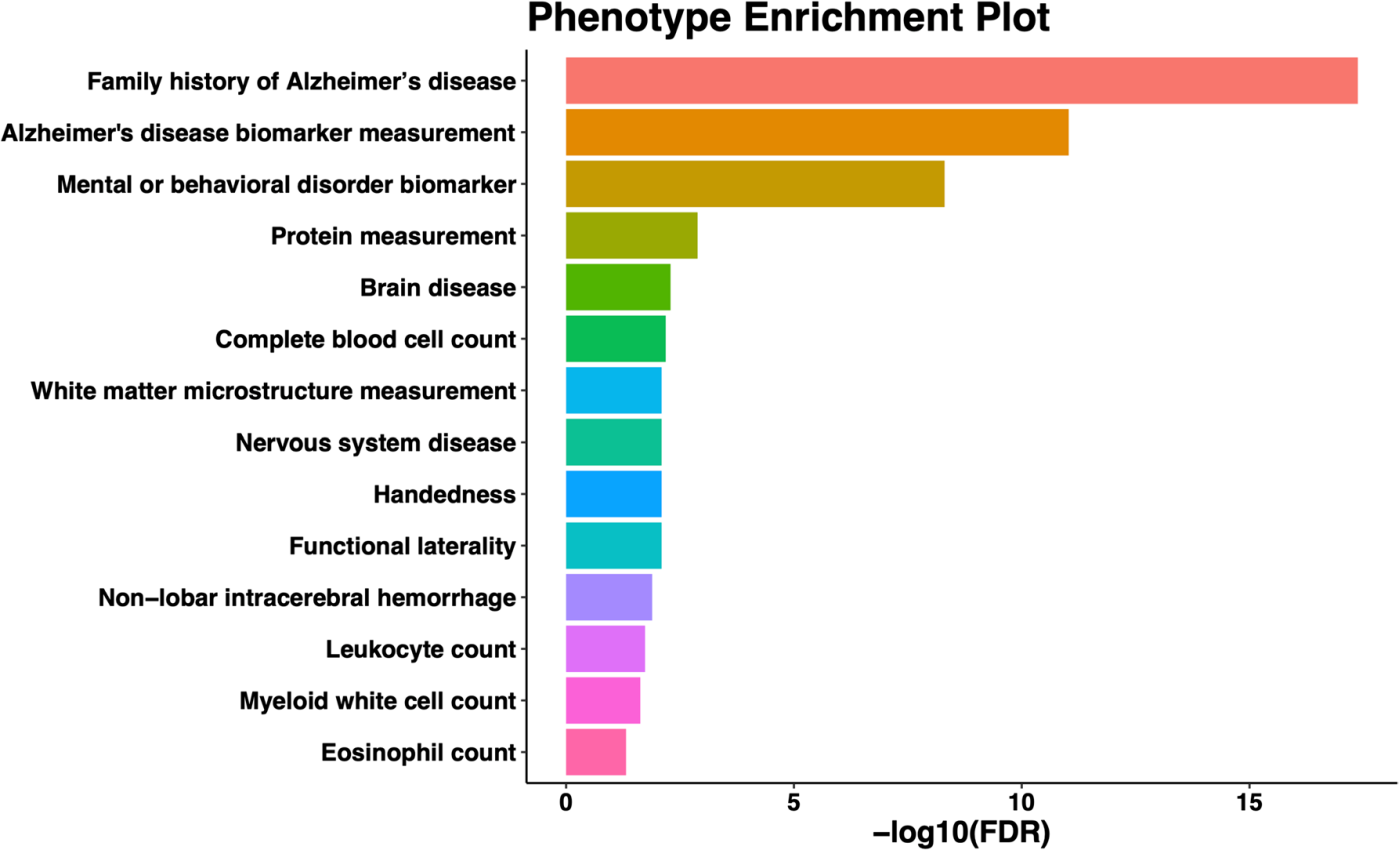
Phenotype enrichment analysis with 141 significant TWAS risk genes by the STRING tool. The -log10 of the false discovery rates (FDR, x-axis) for testing the enrichment of known risk genes of the corresponding phenotype (y-axis) were plotted

By phenotype enrichment analyses conducted by the STRING tool, we found that our identified 141 TWAS risk genes were enriched with 14 phenotypes (Fig. 5), including AD related phenotypes such as family history of AD (false discovery rate, FDR = 4.20e-18), AD biomarker measurement (FDR = 9.30e-12), mental or behavioral disorders (FDR = 4.93e-09), complete blood cell count (FDR = 6.50e-03), functional laterality (FDR = 8.00e-03), white matter microstructure measurement (FDR = 8.00e-03), non-lobar intracerebral hemorrhage (FDR = 1.29e-02), leukocyte count (FDR = 1.85e-02), myeloid white cell count (FDR = 2.35e-02), and eosinophil count (FDR = 4.83e-02). For example, TWAS risk genes *APOC2*, *BIN1*, *SLC24A4*, *BCL3*, *RELB*, and *CLPTM1* are known risk genes for family history of AD, AD biomarker measurement, and mental or behavioral disorders; TWAS risk genes *MAPT*, *WNT3*, and *LINC02210* were related to white matter microstructure measurement; TWAS risk genes *MAPT*, *TUBB*, *PPP5C*, *FCER1G*, *INPP5D*, *NDUFS2*, *NDUFAF6*, and *BCL3* are common risk genes for complete blood cell count, leukocyte count, myeloid white cell count, and eosinophil count.

Interestingly, the enriched phenotypes of functional laterality, intracerebral hemorrhage, white matter microstructure measurement, complete blood cell count, leukocyte count, myeloid white cell count, and eosinophil count have been reported to be associated with AD by previous studies. According to a high-resolution MRI study, lack of the laterality shift in limbic system and early loss of asymmetry in entorhinal cortex could be biomarkers to identify preclinical AD [55]. Several studies found that the risk of dementia increased significantly after intracerebral hemorrhage [56, 57]. A study observed microstructural damage of white matter in AD brains [58]. Chronic inflammation has been proposed as a significant risk factor in AD pathogenesis [59]. Increased levels of complete blood cell count and immune cell count, including leukocyte, myeloid white cell, and eosinophil, have been observed in the brains of AD patients [60–63].

Further, we conducted separate phenotype enrichment analyses by using tissue-specific TWAS risk genes that are of upregulated (with positive TWAS Z-score) and downregulated (with negative TWAS Z-score) groups (Supplemental Figure 7). We found that mental or behavioral disorders and functional laterality were enriched in the upregulated genes in prefrontal cortex, cortex, and whole blood. Additionally, family history of AD was enriched in the upregulated genes in cortex and whole blood, and the downregulated genes in whole blood; AD biomarker measurement was enriched in the downregulated genes in whole blood. No phenotype was found enriched in the downregulated genes in prefrontal cortex and cortex.

## Discussion

In this study, we applied the BGW-TWAS method to the reference GTEx V8 data of three tissues (prefrontal cortex, cortex, and whole blood) to leverage both *cis*- and *trans*-acting eQTL for studying AD dementia. With the latest GWAS summary data of AD dementia [5], we identified 85 significant genes with the prefrontal cortex, 82 significant genes with the cortex, and 76 significant genes with the whole blood reference transcriptomic data. Combining BGW-TWAS *p*-values by ACAT-O, we obtained 141 significant TWAS risk genes of AD dementia, including 35 mapped GWAS risk genes in GWAS Catalog and 34 TWAS risk genes primarily driven by *trans*-eQTL. As expected, most of the genes exhibiting significance due to *trans*-eQTL were not detected by previous GWAS and TWAS which failed to account for *trans*-eQTL. Interestingly, previous studies reported AD-related biological functions for our identified TWAS risk genes that have no *cis*-SNP with significant GWAS *p*-value, such as *NRP2, OTULIN*, *DDR1,* and *TUBB* [48–51].

Through protein-protein interaction network analysis using the STRING tool, we identified network clusters containing mapped GWAS risk genes of AD in GWAS Catalog and our identified TWAS risk genes that are either driven by *trans*-eQTL or/and have no *cis*-SNP with significant GWAS *p*-value. A total of 46 identified TWAS risk genes that are not reported as mapped risk genes in GWAS catalog, are connected with mapped AD GWAS risk genes in the identified protein-protein interaction clusters. Our findings are consistent with previous studies highlighting the critical involvement of *APOC2*, *BIN1*, and *MAPT* in AD [52–54], as evidenced by their extensive connectivity with other mapped GWAS risk genes of AD and TWAS risk genes within the major network cluster. The identified protein-protein interaction networks also underline the important function of genes in the CD20-like family. Since the “text-mining” channel (see Methods) used by the STRING tool identified the majority of the protein-protein interactions among our identified TWAS risk genes, further functional studies by biological experiments are still needed to validate these interactions.

We also identified 14 phenotypes whose known risk genes were enriched in our identified 141 TWAS risk genes. Especially, brain lesions such as functional laterality, intracerebral hemorrhage, and white matter microstructure have been reported to be associated with AD by previous studies [55–58]. Biomarkers such as complete blood cell count, leukocyte count, myeloid white cell count, and eosinophil count were shown to be related to AD [60–63], suggesting a complex interplay among genetic, transcriptomic, metabolic, and inflammatory risk factors in the pathogenesis of AD. Shared risk genes between these biomarkers and AD have also been reported by previous studies. For example, gene *BCL3* and *INPP5D* were reported with pleiotropy effects on AD and inflammation biomarkers [64, 65]. Further biological experiments are still needed to understand the roles of these TWAS risk genes in the biological mechanism of AD.

Nevertheless, our study has several limitations. First, due to the computation burden of running BGW-TWAS tool, we only applied BGW-TWAS to reference GTEx V8 data of three tissues (prefrontal cortex, cortex, and whole blood). Other tissues within and outside the brain such as hippocampus, muscle, and spinal cord, are also known to play crucial roles in the biological mechanisms of AD dementia [66–70]. Failing to consider all available tissues in GTEx V8 data may fail to capture a full spectrum of genetically regulated gene expression associated with AD dementia. Our ongoing work includes further improving the computation efficiency of the BGW-TWAS tool and applying it to all available tissues in GTEx V8.

Second, since samples in the summary-level GWAS data of AD dementia (generated by Bellenguez et. al.) and the GTEx reference dataset are all European ancestries, our TWAS significant gene findings are also limited to the European population. Conducting similar TWAS analyses with reference transcriptomic data and GWAS summary data of other populations would be needed to study the disparity of AD risks in different populations.

Third, the BVSR model employed in the BGW-TWAS method inherently assumes a sparse model implying that only a small number of eQTL have true causal effects on gene expression. Although this assumption can be computationally advantageous, it may not always accurately represent the underlying genetic architecture of complex gene expression quantitative traits. In our future work, we will also investigate using other statistical models for genome-wide TWAS analysis.

## Conclusions

In conclusion, our study highlights the importance of considering both *cis*- and *trans*-eQTL in TWAS analysis as it can help identify significant risk genes that would have been missed by using only *cis*-eQTL. We identified several well-known AD risk genes as well as genes that are not reported in GWAS Catalog but interconnected with known AD risk genes in the same protein-protein interaction networks. As a genome-wide TWAS, our study is the first to utilize both *cis*- and *trans*- eQTLs of multiple tissues for AD risk gene identification. Our results provide further insights into the underlying biological mechanisms of AD dementia and a list of potential gene targets for the development of therapeutics for treating AD dementia.

### Supplementary Information

Supplementary Material 1. 

Supplementary Material 2. 

Supplementary Material 3. 

Supplementary Material 4. 

Supplementary Material 5. 

Supplementary Material 6. 

Supplementary Material 7. 

Supplementary Material 8. 

Supplementary Material 9. 

Supplementary Material 10. 

Supplementary Material 11. 

## Data Availability

The RNAseq transcriptomic and WGS genetic data of GTEx V8 are publicly available from dbGAP (phs000424.v8.p2) and the GTEx portal website (https://gtexportal.org/home/). The summary-level GWAS data of AD dementia was generated by Bellenguez et. al., which are publicly available at https://www.ebi.ac.uk/gwas/studies/GCST90027158. The BGW-TWAS tool is available freely on GitHub (https://github.com/yanglab-emory/BGW-TWAS). The STRING tool is available online (https://string-db.org/). Summary data of our trained gene expression prediction models by BGW-TWAS method and our TWAS results are publicly available on SYNAPSE (10.7303/syn54950908).

## Abbreviations

AD: Alzheimer’s disease
GWAS: Genome-wide association studies
TWAS: Transcriptome-wide association study
BGW-TWAS: Bayesian Genome-wide TWAS
eQTL: Expression quantitative trait loci
GTEx: Genotype-Tissue Expression
ACAT-O: Omnibus aggregated Cauchy association test
GReX: Genetically regulated gene expression
BVSR: Bayesian variable selection regression
EM-MCMC: Expectation-maximization Markov Chain Monte Carlo
PCP: Posterior causal probabilities
WGS: Whole genome sequencing
TPM: Transcripts Per Million
