# Supplementary figures and images for: Bayesian genome-wide TWAS with reference transcriptomic data of brain and blood tissues identified 141 risk genes for Alzheimer’s disease dementia
Source: Alzheimers Res Ther. 2024 Jun 1;16:120. doi: 10.1186/s13195-024-01488-7 (PMC11144322; doi:10.1186/s13195-024-01488-7)

Brain\_Prefrontal\_Cortex   Brain\_Cortex

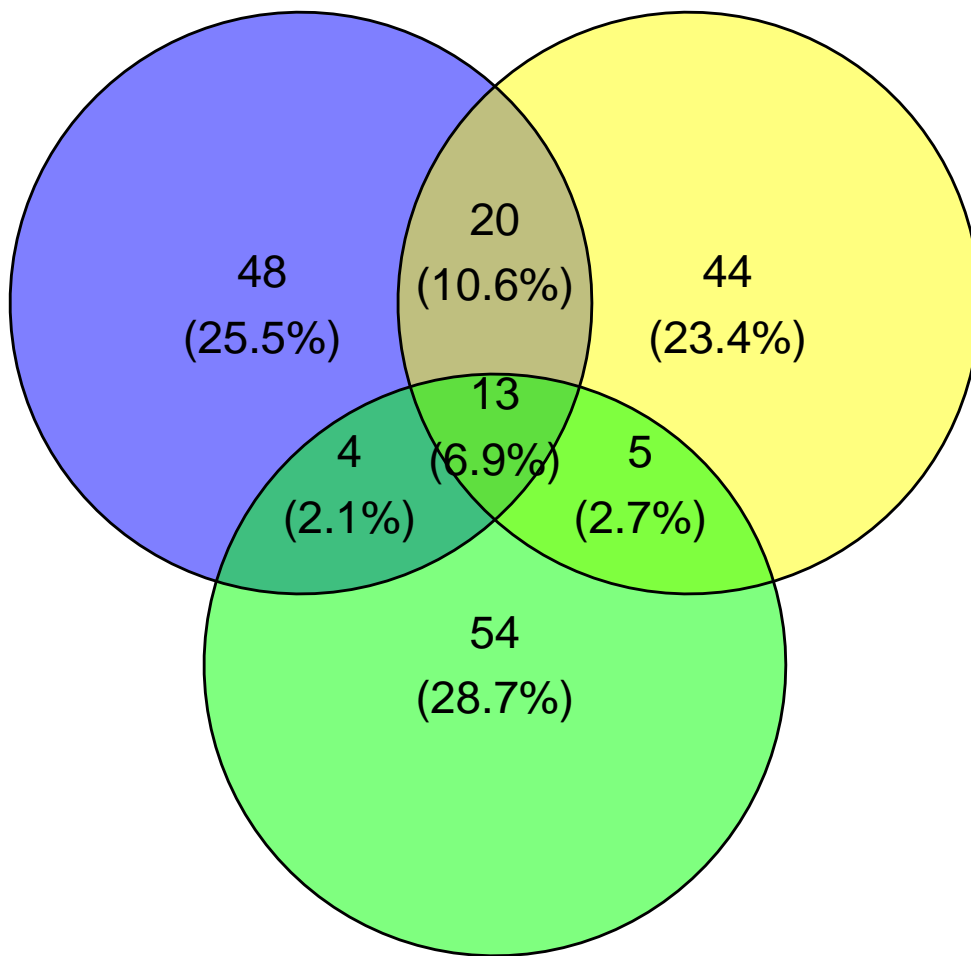

Whole\_Blood

Supplement: Supplementary file 5 — Supplementary Material 5. [file 13195_2024_1488_MOESM5_ESM.pdf]

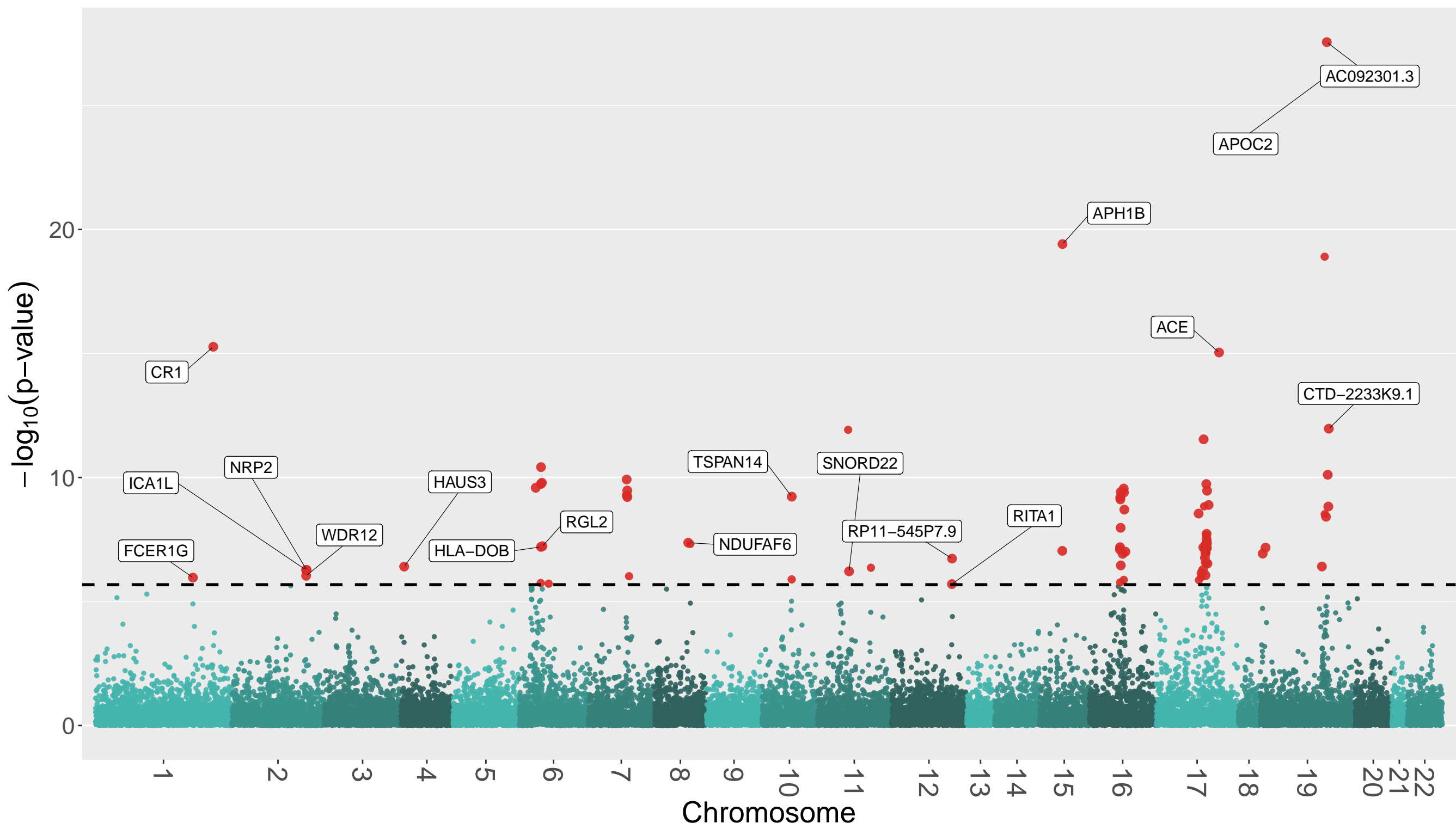

Supplement: Supplementary file 6 — Supplementary Material 6. [file 13195_2024_1488_MOESM6_ESM.pdf]

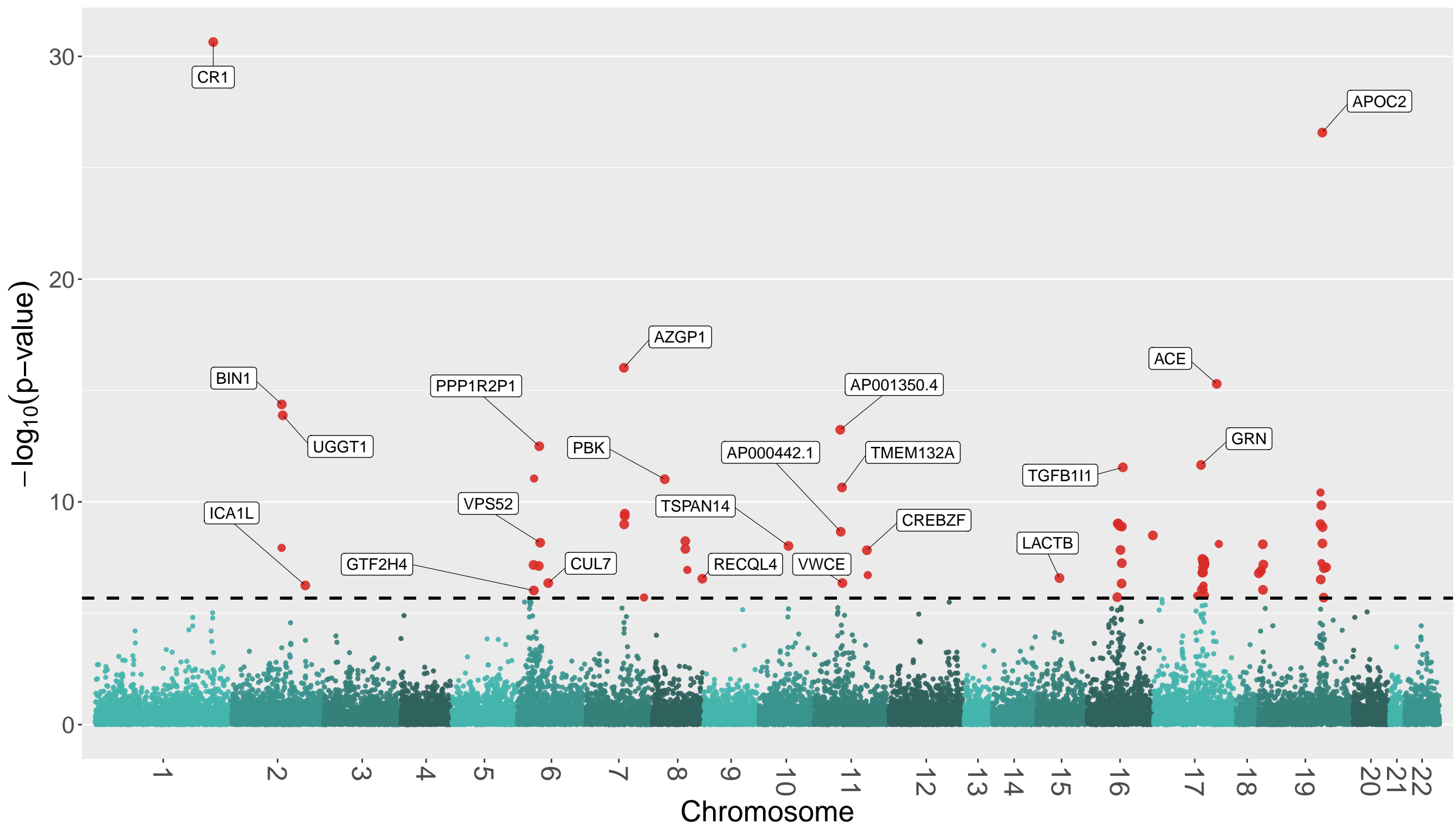

Supplement: Supplementary file 7 — Supplementary Material 7. [file 13195_2024_1488_MOESM7_ESM.pdf]

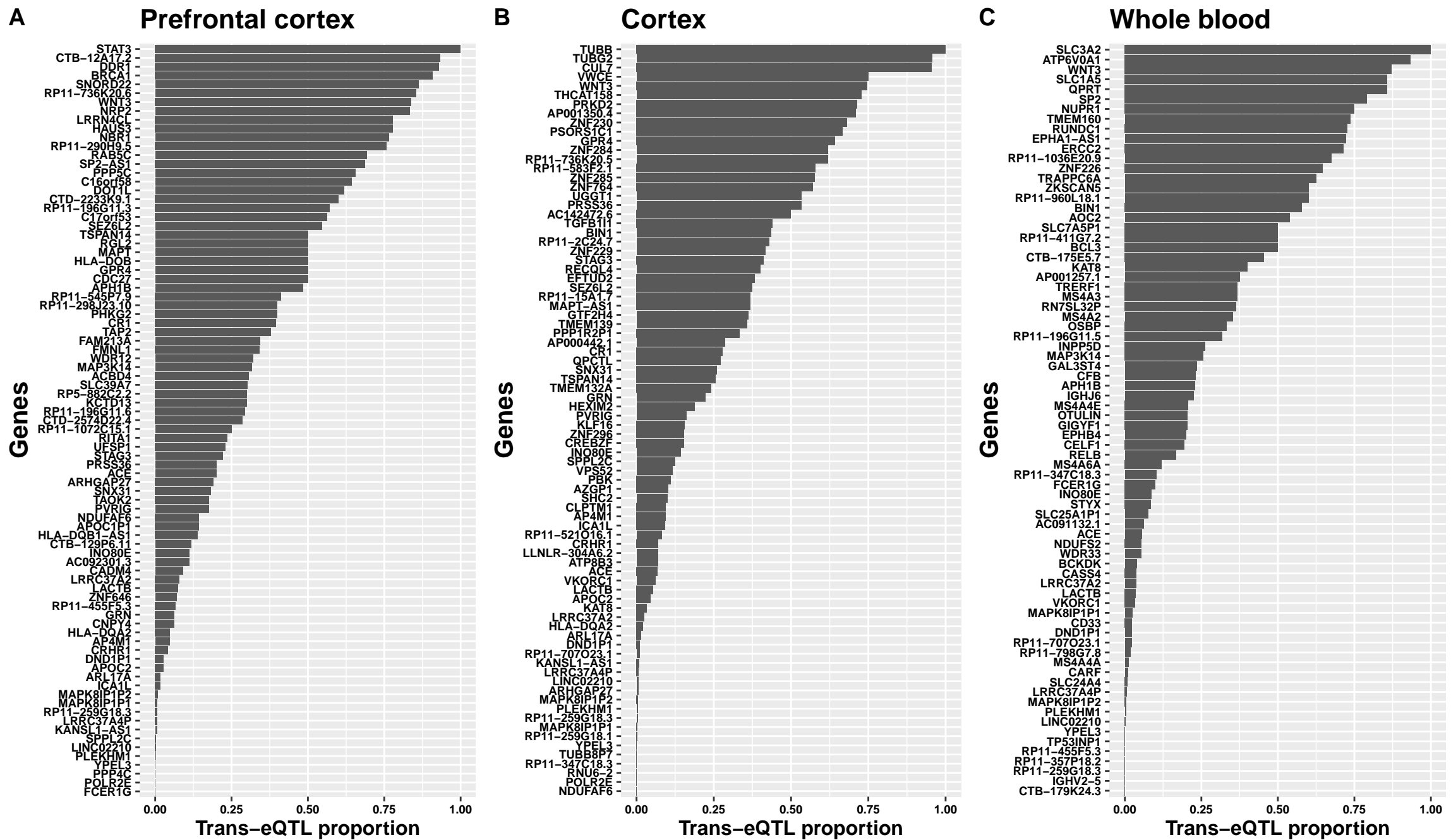

Supplement: Supplementary file 9 — Supplementary Material 9. [file 13195_2024_1488_MOESM9_ESM.pdf]

ACAT\_O

ACAT\_O\_cis\_only

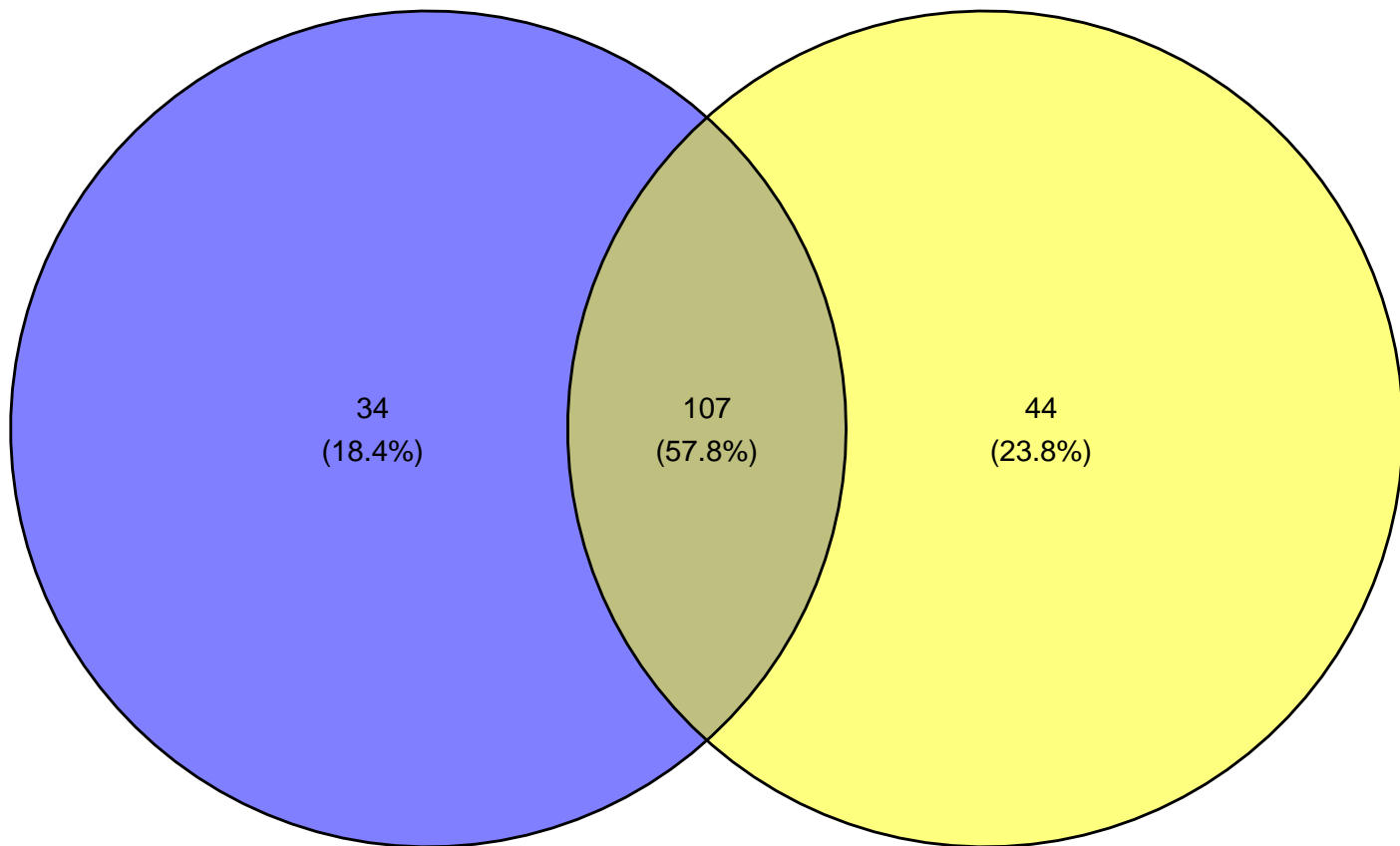

Supplement: Supplementary file 10 — Supplementary Material 10. [file 13195_2024_1488_MOESM10_ESM.pdf]
