# Supplementary material for: Bayesian genome-wide TWAS with reference transcriptomic data of brain and blood tissues identified 141 risk genes for Alzheimer’s disease dementia
Source: Alzheimers Res Ther. 2024 Jun 1;16:120. doi: 10.1186/s13195-024-01488-7 (PMC11144322; doi:10.1186/s13195-024-01488-7)

Phenotype Enrichment Plots of TWAS Risk Genes up/downregulated in the 3 tissues

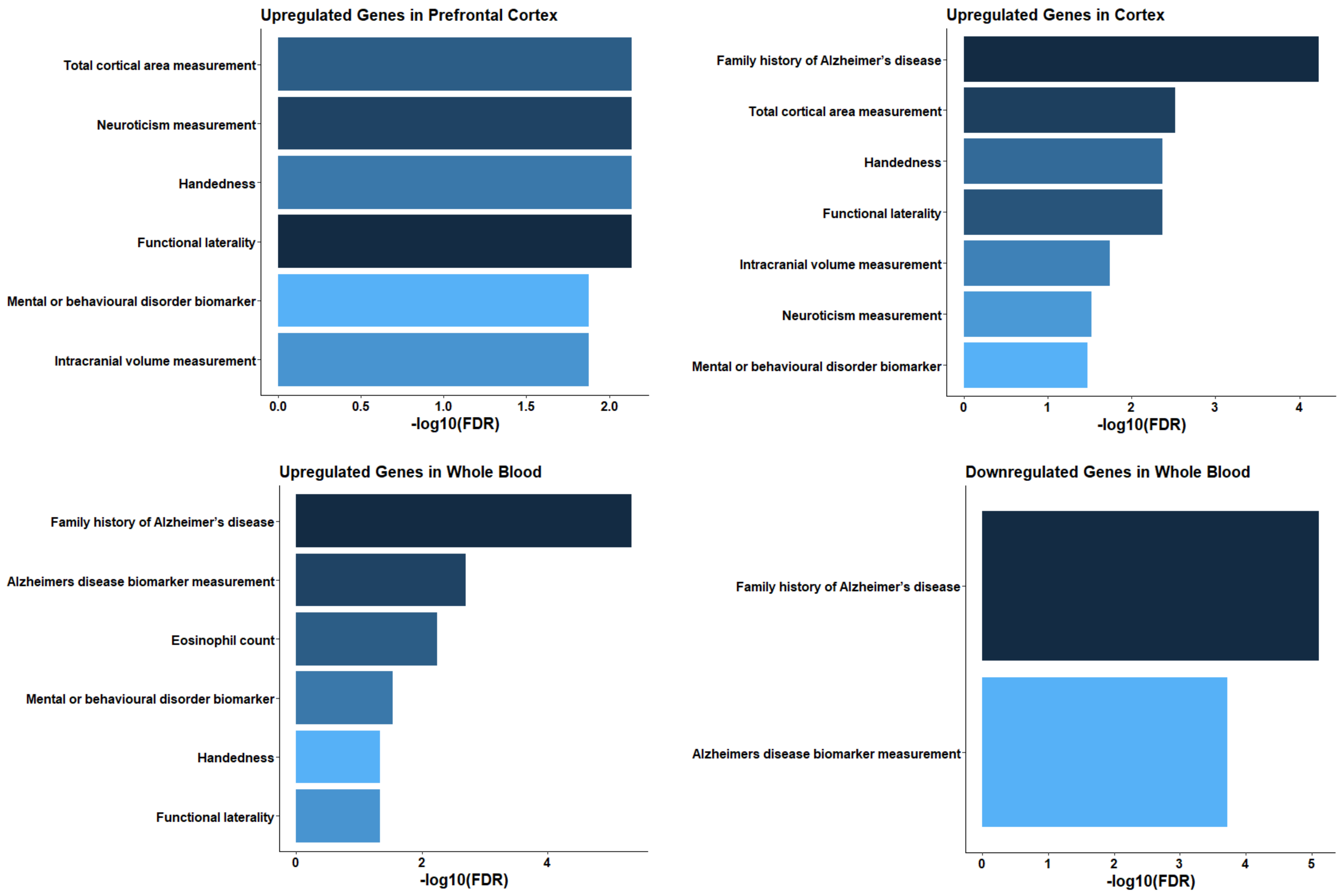

Supplement: Supplementary file 11 — Supplementary Material 11. [file 13195_2024_1488_MOESM11_ESM.pdf]
