# Supplementary material for: Bayesian genome-wide TWAS with reference transcriptomic data of brain and blood tissues identified 141 risk genes for Alzheimer’s disease dementia
Source: Alzheimers Res Ther. 2024 Jun 1;16:120. doi: 10.1186/s13195-024-01488-7 (PMC11144322; doi:10.1186/s13195-024-01488-7)

Supplemental Figure 4: Manhattan plot of BGW-TWAS p-values of whole blood for studying AD dementia. The horizontal dashed line represents the Bonferroni corrected significance threshold of the p-value, given by dividing 0.05 by the total number of test genes. Red dots represent genes that were significant in whole blood. Part of the significant genes that also have significant ACAT-O p-values are labeled.

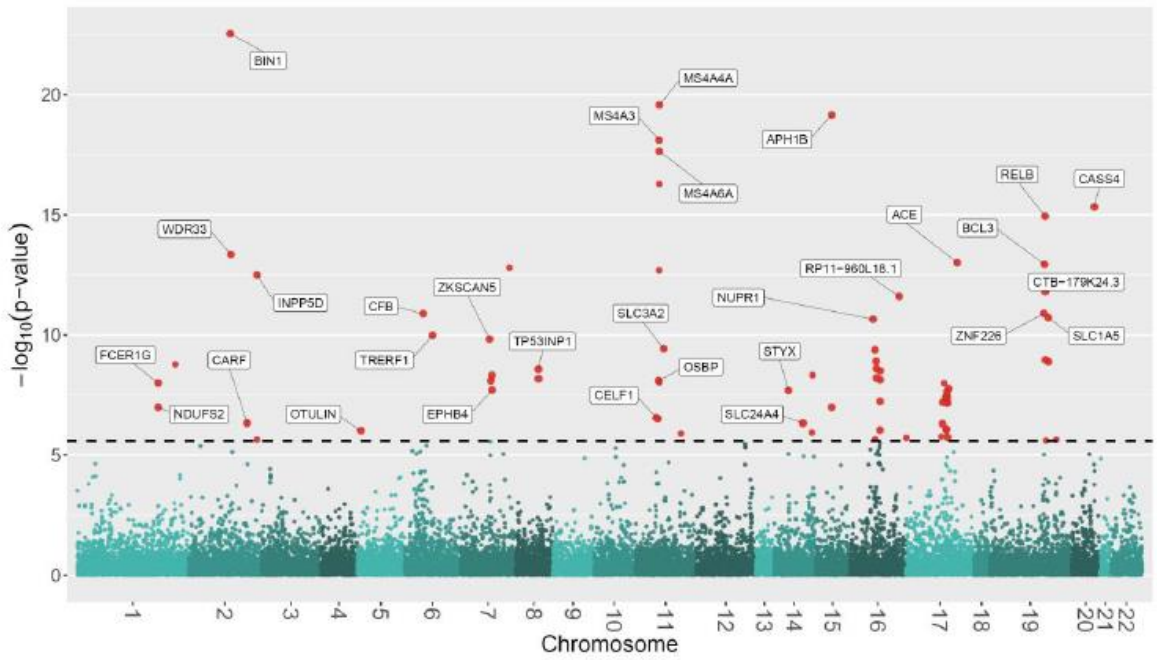

Supplement: Supplementary file 8 — Supplementary Material 8. [file 13195_2024_1488_MOESM8_ESM.pdf]
